# Vitamin E Nicotinate

**DOI:** 10.3390/antiox6010020

**Published:** 2017-03-13

**Authors:** Kimbell R. Duncan, Yuichiro J. Suzuki

**Affiliations:** Department of Pharmacology and Physiology, Georgetown University Medical Center, 3900 Reservoir Road NW, Washington, DC 20057, USA; krd49@georgetown.edu

**Keywords:** tocopherol nicotinate, tocopheryl nicotinate, vitamin E nicotinate

## Abstract

Vitamin E refers to a family of compounds that function as lipid-soluble antioxidants capable of preventing lipid peroxidation. Naturally occurring forms of vitamin E include tocopherols and tocotrienols. Vitamin E in dietary supplements and fortified foods is often an esterified form of α-tocopherol, the most common esters being acetate and succinate. The vitamin E esters are hydrolyzed and converted into free α-tocopherol prior to absorption in the intestinal tract. Because its functions are relevant to many chronic diseases, vitamin E has been extensively studied in respect to a variety of diseases as well as cosmetic applications. The forms of vitamin E most studied are natural α-tocopherol and the esters α-tocopheryl acetate and α-tocopheryl succinate. A small number of studies include or focus on another ester form, α-tocopheryl nicotinate, an ester of vitamin E and niacin. Some of these studies raise the possibility of differences in metabolism and in efficacy between vitamin E nicotinate and other forms of vitamin E. Recently, through metabolomics studies, we identified that α-tocopheryl nicotinate occurs endogenously in the heart and that its level is dramatically decreased in heart failure, indicating the possible biological importance of this vitamin E ester. Since knowledge about vitamin E nicotinate is not readily available in the literature, the purpose of this review is to summarize and evaluate published reports, specifically with respect to α-tocopheryl nicotinate with an emphasis on the differences from natural α-tocopherol or α-tocopheryl acetate.

## 1. Introduction to Vitamin E

Vitamin E refers to a family of compounds that are lipid-soluble antioxidants capable of preventing lipid peroxidation. Naturally occurring forms of vitamin E include four tocopherols (α, β, γ, δ) and four tocotrienols (α, β, γ, δ) [1]. Tocopherols are exclusively synthesized by photosynthetic organisms, and plant-derived oils are the major sources of vitamin E in the human diet. The most common form of tocopherol in the North American diet is γ-tocopherol, the predominant form of vitamin E in corn oils, while the form with the highest biological activity and most common form in European diets is α-tocopherol, found in olive and sunflower oils [2]. Tocotrienols are found in palm oil, barley, oats, and rice bran, and have higher antioxidant activity than tocopherols.

Vitamin E was first discovered in 1922 by Evans and Bishop as “a hitherto unrecognized dietary factor necessary for reproduction” [3]. It was subsequently named by Sure in 1924 and the antioxidant function of vitamin E was identified by Cummings and Mattill in 1931 [4]. In 1936, Evans et al. [5] isolated vitamin E from wheat germ oil, and Karrer et al. [6] synthesized α-tocopherol in 1938. It was first reported to have therapeutic effects in patients with cardiovascular disease by Vogelsang and Shute in 1946 [7]. A relationship between vitamin E deficiency and neurological dysfunction was identified by Binder et al. in 1967 in case studies of steatorrhea [8].

Following these early studies, investigations into the preventative and therapeutic effects of vitamin E supplementation were studied in a variety of diseases including atherosclerosis, hypertension, angina, cancer, inflammation, hematologic disorders, diabetes, purpura, Alzheimer’s disease, Parkinson’s disease, and ataxia with vitamin E deficiency. Generally, supplementation appears to be beneficial in diseases in which vitamin E deficiency is being remedied, and does not appear to be beneficial in supraphysiological doses in patients with normal vitamin E levels; indeed, it may result in deleterious prooxidant effects and increase mortality rates. Miller et al. found in their meta-analysis of human trials that there was no change in the all-cause mortality associated with vitamin E supplementation in general, but calculated a risk ratio of 1.04 for studies in which patients received a “high dose” of vitamin E supplementation (defined as >400 IU/day) [9]. However, the meta-study by Abner et al. found no effect (risk ratio of 1.00) on all-cause mortality for vitamin E supplementation at doses up to 5500 IU/day [10].

Biochemically, α-tocopherol functions as a chain-breaking antioxidant that interrupts the propagation of reactive oxygen species through lipid membranes by scavenging lipid peroxyl radicals [1]. The rate of reaction between α-tocopherol and lipid peroxyl radicals is several orders of magnitude greater than the reaction between lipid peroxyl radicals and lipid molecules [11]. During the chain-breaking reaction, α-tocopherol forms a free radical. This α-tocopherol radical can react rapidly with another peroxyl radical, thus terminating two peroxidation chains, or it can be reduced by vitamin C or ubiquinol back to α-tocopherol [12].

In addition to the antioxidant activity of α-tocopherol, in vitro studies have demonstrated that it can decrease the activity of several enzymes including nicotinamide adenine dinucleotide phosphate (NADPH) oxidases, protein kinase C, and phospholipase A2 [11]. It has also been demonstrated to regulate the expression of several genes including those for α-tropomyosin, collagenase, α-tocopherol transfer protein, low-density lipoprotein (LDL) scavenger receptors, class A scavenger receptor (SR-A), and CD36 as well as intercellular cell adhesion molecule-1 [13]. Suzuki and Packer demonstrated that two vitamin E esters, α-tocopheryl acetate and α-tocopheryl succinate, inhibited the activation of nuclear factor (NF)-κB [14]. 

The natural form of α-tocopherol is easily oxidized and relatively unstable; as such, esters of α-tocopherol have been developed for supplementation and use in cosmetics. Esterified forms of α-tocopherol are resistant to oxidation and have a longer shelf life than natural forms of α-tocopherol. Upon ingestion, the esters are removed by the esterases at the lumen of the intestine, thus resulting in free α-tocopherol with the same bioavailability as natural α-tocopherol. As such, esterified α-tocopherols are sometimes referred to as prodrug forms of α-tocopherol. The most common form of ester is α-tocopheryl acetate. However, both supplements and cosmetics may include other vitamin E esters such as α-tocopheryl succinate and a molecule called α-tocopheryl nicotinate [15]. While the acetate and succinate forms of vitamin E esters are widely known, vitamin E nicotinate has not been well recognized.

## 2. Rationale and Purpose of This Review

**Figure 1:**
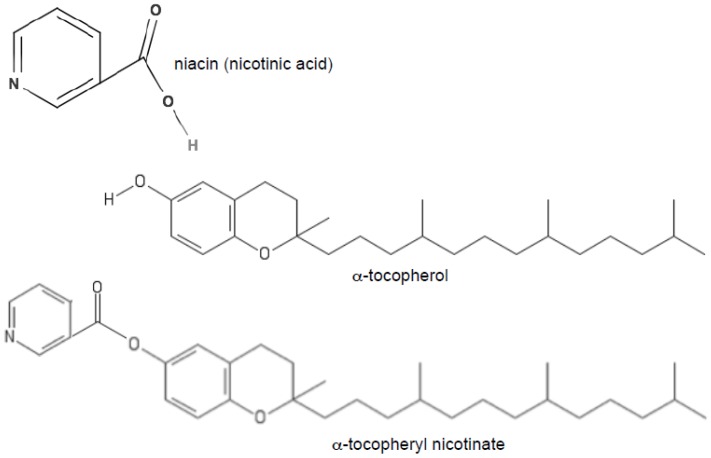
Chemical structures of niacin (nicotinic acid), α-tocopherol, and α-tocopheryl nicotinate.

**Figure 2:**
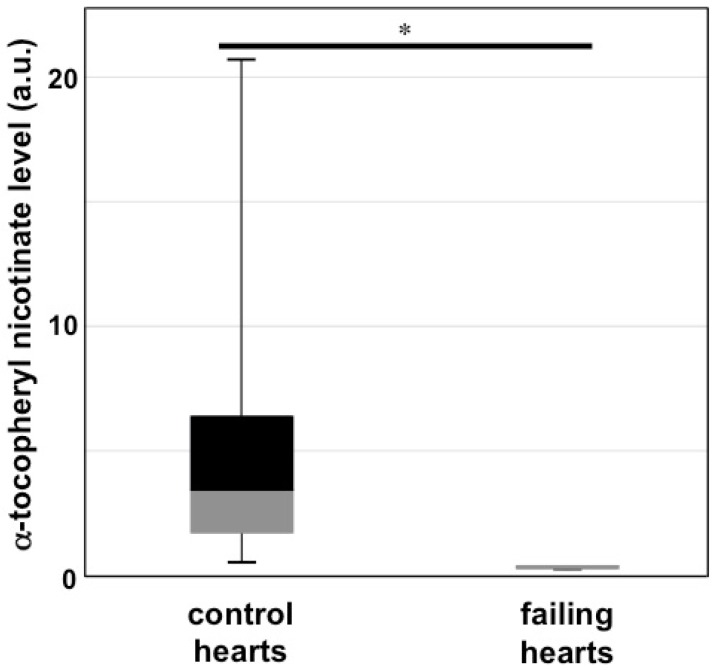
Levels of α-tocopheryl nicotinate in the heart of healthy control rats and rats with heart failure. Pulmonary arterial hypertension-induced right-sided heart failure was generated by administering ovalbumin and SU5416 to Sprague-Dawley rats [16]. Homogenates of right-heart ventricular tissues were injected into a reverse-phase column of an Acquity ultra-performance liquid chromatography (UPLC) system. Mass spectrometry (MS) was performed using a quadrupole-time-of-flight mass spectrometer. The box-and-whisker plot represents the levels of the metabolite corresponding to α-tocopheryl nicotinate (*m*/*z* 536.4077) in arbitrary units (a.u.). * denotes significant difference between each other at *p* < 0.05.

**Figure 3:**
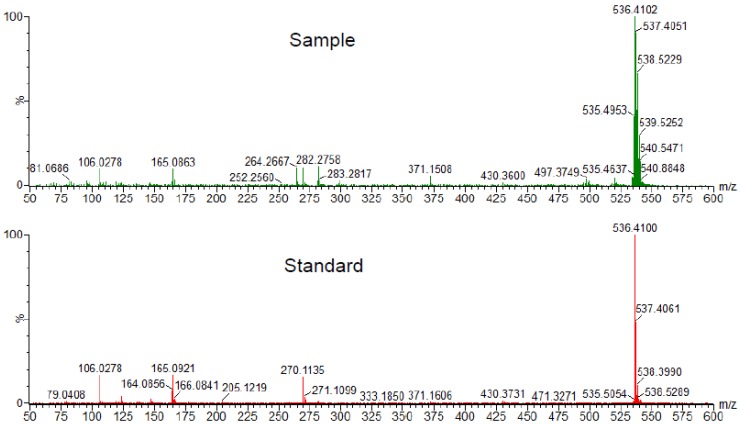
Confirmation of the α-tocopheryl nicotinate peak. Results of metabolomics experiments using UPLC/MS were confirmed by time-of-flight/time-of-flight (TOF/TOF) tandem mass spectrometry. Both healthy control rat right-ventricle homogenate samples and α-tocopherol nicotinate (purchased from Sigma-Aldrich, St. Louis, MO, USA) used as a standard exhibited the *m*/*z* peak at 536.410.

Vitamin E nicotinate is an ester of vitamin E and niacin (vitamin B_3_). Figure 1 shows the chemical structures of α-tocopherol, niacin (nicotinic acid), and α-tocopheryl nicotinate. We came across α-tocopheryl nicotinate only when we performed metabolomics analysis in a list of differentially expressed metabolites between controls and rats with heart failure. In order to understand the mechanism of right-sided heart failure induced by pulmonary arterial hypertension, we subjected Sprague-Dawley rats to the SU5416/ovalbumin model of pulmonary arterial hypertension [16]. The right ventricles of these rats had severe damage to cardiomyocytes and exhibited severe fibrosis [17]. We then took the right ventricles from these rats as well as from healthy control rats and submitted them to metabolomics analysis. Among a number of metabolites that were found to have at least 2-fold changes in their contents with a probability of *p* ≤ 0.05, a metabolite with a molecular mass of 536.4077 was found to be 28-fold different with *p* = 0.02859. These results suggested that, compared with the control, the right ventricles of rats with pulmonary arterial hypertension and right-sided heart failure were found to have 28-fold less α-tocopheryl nicotinate (Figure 2). To confirm the metabolomics experiments performed with ultra-performance liquid chromatography and quadrupole-time-of-flight mass spectrometry, we performed mass spectrometry–mass spectrometry analysis. The results showed that the metabolite that was found to be decreased 28-fold in rats with right-sided heart failure had exactly the same mass as α-tocopheryl nicotinate standard from Sigma-Aldrich (St. Louis, MO, USA) (Figure 3). These rats were fed Laboratory Rodent Diet 5001 from LabDiet (St. Louis, MO, USA), which contains dl-α-tocopheryl acetate as a source of vitamin E. Thus, α-tocopheryl nicotinate is likely formed endogenously from dietary vitamin E and niacin. These results demonstrated that (i) α-tocopheryl nicotinate endogenously occurs in rat hearts and (ii) this cardiac α-tocopheryl nicotinate is dramatically reduced in heart failure. These findings highlight the possible pathophysiological importance of vitamin E nicotinate.

Since we were not aware of α-tocopheryl nicotinate, we performed PubMed and Google searches of α-tocopheryl nicotinate. However, the term “tocopheryl nicotinate”, “tocopherol nicotinate”, and “vitamin E nicotinate” were largely only found as a supplement in cosmetics. Moreover, no review articles have been published on this topic. Thus, the purpose of this review is to summarize and evaluate the literature specifically with respect to α-tocopheryl nicotinate with an emphasis on its differences from natural α-tocopherol or α-tocopheryl acetate.

## 3. Cosmetic Applications

The safety report issued by the Cosmetic Ingredient Review Expert Panel in 2002 demonstrates that α-tocopheryl nicotinate is used relatively infrequently (<0.3% of applications using α-tocopherol or derivatives thereof) in cosmetic applications (Table 1). This is most likely due to the lower uptake of α-tocopherol by skin with the nicotinate ester compared with the acetate ester and the lower UV protection afforded by the nicotinate esterified form relative to the acetate-esterified form [15].

## 4. Trademarks

Eisai Co., Ltd. (Kamisu, Ibaraki-ken, Japan) markets α-tocopheryl nicotinate under the trademark Juvela N^TM^ and labels it as a “microcirculation activator”. It claims clinical efficacy for hypertension, hyperlipidemia, and peripheral circulatory disturbances. It further claims treatment to result in the improvement of lipid metabolism, enhancement of microcirculation, strengthening of blood vessels, inhibition of platelet aggregation, and restoration of arterial oxygen partial pressure. Pfizer markets α-tocopheryl nicotinate under the trademark Renascin^TM^ [18].

## 5. Intake and Metabolism

The U.S. Food and Drug Administration’s Recommended Daily Allowance (RDA) for α-tocopherol is 15 mg (equivalent to 22.5 IU) and the daily tolerable upper intake limit is 1000 mg (equivalent to 1500 IU) for adults. Troesch et al., in their review of nutritional surveys, reported that 75% of adults in both the United States and the United Kingdom do not meet the RDA level of intake [19]. Peter et al., in their review of global α-tocopherol status, estimated that the median intake of α-tocopherol is 6.2 mg/day and a median serum α-tocopherol concentration is 22.1 μM globally [20]. 

Peroxide-induced hemolysis has been demonstrated to occur at serum α-tocopherol concentrations below 12 μM and clinical vitamin E deficiency symptoms such as peripheral neuropathy, ataxia, skeletal myopathy, and retinopathy occur at serum α-tocopherol concentrations below 8 μM. In subjects fed a vitamin E-deficient diet, the supplementation of 12 mg/day was sufficient to achieve a serum α-tocopherol concentration of 12 μM. The urine excretion of α-carboxyethyl hydroxychroman—a metabolite produced by a cytochrome P450 (CYP) enzymatic metabolism of α-tocopherol—increases, while exhaled air concentrations of pentane, a marker for oxidative stress, fall at serum concentrations of α-tocopherol above 30 μM, pointing to broader health benefits at this higher serum concentration [20].

The studies by Gallo-Torres et al. demonstrated that higher concentrations of α-tocopherol appear in the lymphatic system of rats treated with α-tocopheryl nicotinate than when rats are treated with α-tocopheryl acetate, and demonstrated that bile salts and pancreatic juice are required for the intestinal absorption and lymphatic presentation of α-tocopherol [21,22]. They noted that between 76% and 90% of radiolabeled α-tocopheryl nicotinate is converted into unesterified α-tocopherol before appearing in the lymph. In a follow-up study, the Gallo-Torres group compared the appearance of α-tocopherol in blood, liver, spleen, and kidney tissues after the oral administration of radio-labeled α-tocopheryl nicotinate or α-tocopheryl acetate [23]. They found that tissue concentrations were higher in the α-tocopheryl acetate-treated group at every time point between 3 h and 48 h after treatment, except the 12 h time point. They concluded that both α-tocopheryl nicotinate and α-tocopheryl acetate were readily converted into free α-tocopherol following oral administration, although larger concentrations of α-tocopheryl nicotinate were found in the blood, suggesting that more α-tocopheryl nicotinate escapes hydrolysis in the gastrointestinal tract compared with α-tocopheryl acetate.

Nakamura et al. investigated the intestinal absorption of 12 different α-tocopherol esters in rats [24]. They also demonstrated that α-tocopheryl nicotinate was hydrolyzed more slowly than α-tocopheryl acetate and that the hydrolysis of esters was not a prerequisite for intestinal absorption.

Hasegawa et al. investigated the effect of food intake on the absorption of α-tocopheryl nicotinate in beagle dogs and healthy human subjects [25]. They found that maximum blood concentrations of α-tocopheryl nicotinate were 5-fold higher in non-fasted beagle subjects than in fasted beagle subjects and 32-fold higher in non-fasted human subjects than in fasted human subjects. This group did not measure α-tocopherol concentrations following supplementation but rather the esterified form; however, they noted that their results are consistent with those of Gallo-Torres et al., since food intake stimulates the flow of bile and pancreatic secretions necessary for the lipid–bile micelle formation required for absorption.

Suzuki and Nakamura investigated the metabolism of the nicotinic acid moiety of α-tocopheryl nicotinate [26]. They found that the main metabolite of the tocopherol moiety from α-tocopheryl nicotinate found in plasma and red blood cells was tocopheryl quinone and that the main metabolite of the nicotinic acid moiety in plasma and red blood cells was nicotinamide. They also found α-tocopheryl nicotinate in red blood cells. The group noted that the main urinary metabolite of α-tocopherol-treated rats was nicotinic acid, whereas the metabolite of nicotinic acid-treated rats was nicoturinic acid. They also noted that the rate of the excretion of the nicotinic acid moiety metabolites was approximately twice as fast for the nicotinic acid-treated group compared with the α-tocopheryl nicotinate-treated group, concluding that the two compounds follow different metabolic pathways. They proposed that α-tocopheryl nicotinate may be an effective carrier of nicotinic acid to gradually supply the NAD pathway.

Funakoshi et al. compared the absorption of α-tocopherol from α-tocopheryl nicotinate (Juvela N) supplementation in patients with chronic pancreatitis versus healthy controls [27]. Chronic pancreatitis patients had significantly lower fasting serum levels of free α-tocopherol relative to healthy controls. The chronic pancreatitis patients were divided into two groups, those with pancreatic calcification and those without calcification. The fasting α-tocopherol levels between the two groups was not significantly different, but the rate of absorption and hydrolysis of α-tocopheryl nicotinate was lower in patients with pancreatic calcification, although the absorption of each pancreatitis group was not statistically significantly different to that for healthy controls. They demonstrated that the absorption of α-tocopheryl nicotinate was enhanced by the coadministration of a digestive enzyme, pancreatin. This finding is consistent with the findings of others [25] that the absorption of α-tocopheryl nicotinate is significantly enhanced when administered with food compared with fasting. The weakness of this study was that only nicotinate esters were studied, and no comparison against acetate esters was made.

## 6. Rheology

Koyama and Araiso studied the effects of α-tocopheryl nicotinate on the microdynamics of phospholipid molecules in the erythrocytes of healthy human subjects [28]. They observed that membrane viscosity declined in treated (oral 400 mg/day α-tocopheryl nicotinate for 1 month) subjects relative to controls. However, they found that α-tocopherol concentration increased in erythrocyte membranes following treatment that, according to prior research in vitro, should correspond to a decrease in membrane fluidity. They hypothesized that the decrease in membrane viscosity was due to a change in the constituents of phospholipids in the membranes of the erythrocytes of treated subjects. No comparison was made with the effects of the treatment of the other esters of α-tocopherol.

Chung et al. [29] studied the effects of α-tocopheryl nicotinate treatment on the hemorheological properties and retinal microcirculation of seven human female patients with non-insulin-dependent diabetes mellitus (type 2 diabetes). Diabetic patients frequently present abnormalities in blood rheology, attributed to an increase in the viscosity of whole blood and plasma and red blood cell rigidity, at least partly caused by the oxidative stress of erythrocyte membranes. In this study, they observed a significant improvement in blood viscosity, red cell deformability, and retinal capillary blood flow, but not in plasma viscosity and red blood cell rigidity from treated patients (oral 900 mg/day for 3 months). They noted that no significant changes in total protein or lipoprotein content were observed after 3 months of treatment and hypothesized that the rheological improvements were primarily the result of improved red cell deformity. No controls or other tocopherol esters were included in the study and the number of subjects was small.

Chung et al. [30] investigated the effects of α-tocopheryl nicotinate treatment on reducing lipid peroxidation stress and improving the hemorheological properties of erythrocyte membranes in type 2 diabetic patients with retinopathy [30]. Thirteen patients (8 male, 5 female) were treated for 3 months with the oral administration of α-tocopheryl nicotinate (900 mg/day). No significant changes in hemoglobin contents, hematocrit, total proteins, or lipoprotein content were observed after the 3-month treatment. However, blood viscosity was significantly reduced and red blood cell deformity significantly improved following treatment, consistent with prior studies. They observed a significant reduction in the malondialdehyde of red blood cell membranes corresponding with reduced oxidative stress, but malondialdehyde levels remained above those observed in healthy controls (8 age- and weight-matched healthy subjects). No other tocopherol esters were evaluated.

In a series of studies, Kamimura compared the effects of α-tocopheryl nicotinate with those of α-tocopheryl acetate on skin microcirculation in patients with microcirculation disturbances [31,32]. In the first study, a total of 10 patients were subject to the cross-administration of α-tocopheryl nicotinate and/or α-tocopheryl acetate in combination with nicotinic acid (400 mg/day α-tocopheryl nicotinate or 400 mg/day α-tocopheryl acetate with 80 mg/day nicotinic acid). The treatment regimen consisted of 2 weeks of one treatment followed by 2 weeks of the other, and then followed by 2 weeks of the first treatment. The effect of treatments on microcirculation was measured by changes in the mean rewarming time within a cooling–rewarming test in which a patient’s hands were soaked (up to the wrist) in water, maintained at 15 °C for 5 min, and the time for skin temperature (measured at two points) to rise to 25 °C after removal is determined. They demonstrated that α-tocopheryl nicotinate had a stronger effect in reducing the mean rewarming time than the combination of α-tocopheryl acetate and nicotinic acid, and that both treatments had a significant effect on the mean rewarming time. In another study, eight patients who had previously been treated with α-tocopheryl acetate (400 mg/day for 4–8 weeks) but had shown little or no improvement were switched to α-tocopheryl nicotinate treatment (400 mg/day) for 2 weeks. The mean rewarming time was reduced in all patients. In a further study, 18 patients were subject to the cross-administration of α-tocopheryl nicotinate and α-tocopheryl acetate. The mean rewarming time decreased more significantly following the administration of α-tocopheryl nicotinate and, in most cases, increased when treatment was switched back to α-tocopheryl acetate. Ten patients were subject to the cross-administration of α-tocopheryl nicotinate and α-tocopheryl acetate with nicotinic acid. As with the cross-administration of the nicotinate ester and acetate ester, the mean rewarming time decreased more significantly following the administration of α-tocopheryl nicotinate and, in most cases, increased when treatment was switched back to α-tocopheryl acetate with nicotinic acid. They concluded that α-tocopheryl nicotinate is more effective in reducing the mean rewarming time than α-tocopheryl acetate or α-tocopheryl acetate in combination with nicotinic acid, and that the effect of α-tocopheryl nicotinate is not due to the synergistic effects of α-tocopherol and nicotinic acid but rather due to the independent effect of α-tocopheryl nicotinate on the microcirculatory system. Similarly, the authors hypothesized that the greater effectiveness of α-tocopheryl nicotinate may be due to its slower rate of hydrolysis.

## 7. Cardiovascular

Questions regarding the role of LDL oxidation in atherosclerosis and cardiovascular disease have led to many groups studying the effects of vitamin E supplementation on serum cholesterol and LDL concentrations, cardiovascular disease, and hypertension [1]. An early epidemiological study by Gey and Puska demonstrated that plasma concentrations of vitamin E were inversely correlated with the incidence of ischemic heart disease in 12 different European populations [33]. However, in aggregate, clinical studies have not demonstrated consistently the benefit of vitamin E supplementation in the prevention of cardiovascular disease; in fact, some studies have raised the possibility of the increased risk of heart failure and hemorrhagic stroke and an increase in total mortality [4].

The Cambridge Heart Antioxidant Study (CHAOS) in 2000 patients with coronary atherosclerosis demonstrated that vitamin E supplementation (400–800 IU/day) over 2 years significantly reduced incidences of cardiovascular death and myocardial infarction [34]. The MRC/BHF Heart Protection Study Collaborative Group concluded that vitamin E supplementation did not reduce the risk of death or incidence of heart attacks or strokes in a randomized trial of 20,536 people at increased risk of heart disease [35]. The Antioxidant Supplementation in Atherosclerosis Prevention (ASAP) study (520 subjects) demonstrated that combined supplementation with vitamin C and vitamin E (α-tocopheryl acetate) significantly slowed the rate of progression of atherosclerosis in hypercholesterolemic patients [36].

In two different studies, Igarishi et al. compared the effects of α-tocopheryl nicotinate and α-tocopheryl acetate in rat models of hypertension [37,38]. They demonstrated that treatment with tocopherol esters reduces the progression of hypertension and protects animals from myocardial fibrosis, pulmonary edema, weight loss, and death. They concluded that α-tocopheryl nicotinate is 5 times more potent than α-tocopheryl acetate in these antihypertensive effects.

Iino et al. conducted a controlled, double-blind trial of Juvela-N (α-tocopheryl nicotinate) versus the placebo for the relief of subjectively assessed symptoms in patients with hypertension and cerebral atherosclerosis [39]. A total of 94 subjects were given either 600 mg/day of α-tocopheryl nicotinate or 600 mg/day of the inactive placebo, administered orally in six capsules, for 4 to 6 weeks, and 89 subjects completed the trial (44 in the α-tocopheryl nicotinate-treated group and 45 in the placebo group). They demonstrated that symptoms improved in the α-tocopheryl nicotinate treatment group compared with the placebo group. 

Hidiroglou et al. [40] examined the effects of different tocopherol esters on serum concentrations of triglycerides, total cholesterol, and high-density lipoprotein (HDL)-cholesterol in 40 wether lambs. The animals were divided into seven treatment groups of five animals each and one placebo group. Different ester forms of α-tocopherol were provided over 2 months in equimolar amounts equivalent to 300 mg/lamb/day of α-tocopheryl acetate. While there were significant differences in free serum α-tocopherol levels between the different esters, there were no significant treatment effects for any of the tocopherol ester forms for total cholesterol, triglycerides, or HDL-cholesterol.

Platelet aggregation plays a central role in thrombosis and the pathogenesis of atherosclerosis. In an in vitro study comparing the effects of α-tocopheryl nicotinate with those of α-tocopheryl acetate on hydrogen peroxide-induced platelet aggregation, Higashi and Kikuchi [41] demonstrated that α-tocopheryl nicotinate was more effective than α-tocopheryl acetate in suppressing hydrogen peroxide-induced platelet aggregation. ADP-induced platelet aggregation was not suppressed by α-tocopheryl acetate but α-tocopheryl nicotinate had a suppressive effect. The lipid peroxidation of platelets by hydrogen peroxide was significantly reduced by α-tocopheryl nicotinate but not α-tocopheryl acetate. In a separate in vitro study, Svensson and Oki [42] demonstrated that α-tocopheryl nicotinate and α-tocopheryl acetate are more effective inhibitors than free α-tocopherol in inhibiting platelet aggregation induced by arachidonic acid and collagen. They noted that the nicotinate ester was up to 5 times more potent than the acetate ester and up to 18 times more potent than the unesterified tocopherol. The significance of these in vitro studies is questionable since metabolism studies have demonstrated that virtually all α-tocopheryl acetate and most of α-tocopheryl nicotinate are hydrolyzed in the intestinal tract prior to absorption. It has been demonstrated in several studies that α-tocopheryl nicotinate does appear in the lymph and some tissues after oral administration, suggesting it may be available to inhibit platelet aggregation in the manner suggested in these in vitro studies.

Noma et al. [43] evaluated the effects of α-tocopheryl nicotinate supplementation (600 mg/day) on serum lipoprotein(a) levels in 28 hyperlipidemic patients. After 2 months of α-tocopheryl nicotinate treatment, serum lipoprotein(a) levels significantly declined in patients with initial lipoprotein(a) levels ≥18 mg/dL. Serum lipids, lipoproteins, and apolipoproteins, other than lipoprotein(a), tended to increase in response to α-tocopheryl nicotinate.

Schlieper and Tawfil [18] studied the effect of α-tocopheryl nicotinate (Renascin^TM^), α-tocopherol, and dodecanoic acid on the inotropic action of ouabain and digoxin in pigs. They observed that the nicotinate ester and dodecanoic acid significantly reduced the inotropic effect of digoxin but not ouabain. Arrhythmias induced by both glycosides were suppressed by all three compounds, but α-tocopheryl nicotinate had the greatest antiarrhythmic activity. Nicotinic acid did not demonstrate antiarrhythmic effects.

## 8. Immune Function

Vitamin E deficiency impairs both humoral and cell-mediated immune responses [44]. Supplementation studies have demonstrated the significant enhancement of cell-mediated immunity in elderly patients [45]. The mechanisms of this improvement include enhanced lymphocyte proliferation and interleukin (IL)-2 production and decreased prostaglandin E_2_ (PGE_2_) production through cyclooxygenase (COX) modulation.

Moriguchi et al. [46] demonstrated that vitamin E enhanced T-cell differentiation in the thymus, while vitamin E supplementation improved cellular immunity that decreases with aging in spontaneously hypertensive rats (SHR) and expedited the recovery of cellular immunity following X-ray irradiation. Moriguchi and Itoh [47] studied the mechanisms by which T-cell differentiation in the thymus was enhanced in normotensive rats fed high α-tocopheryl nicotinate diets (585 mg/kg all-*rac*-α-tocopheryl nicotinate). They concluded that α-tocopheryl nicotinate enhanced thymic epithelial cell function and binding capacity, which induced a significant increase in the proportion of CD4^+^ T-cells. The binding of thymic epithelial cells to immature T-cells is the first step in T-cell differentiation in the thymus. Adhesion molecules lymphocyte function-associated antigen 1 (LFA-1) and intercellular adhesion molecule 1 (ICAM-1) are important mediators of thymic epithelial cell adhesion to immature T-cells, and the expression of ICAM-1 was enhanced following supplementation with α-tocopheryl nicotinate. They concluded that the effect of α-tocopheryl nicotinate supplementation was not the result of the enhanced function of macrophages.

Inagaki et al. [48] compared the effects of α-tocopheryl acetate and α-tocopheryl nicotinate on IgE antibody generation in mice. Both esters inhibited IgE antibody formation in mice challenged with dinitrophenylated ascaris protein in alum in a non-dose-dependent manner. The nicotinate ester had slightly greater potency. Tanaka et al. [49] compared the effects of α-tocopheryl nicotinate and α-tocopheryl acetate on the humoral immune response to hamster erythrocytes in mice (antigen challenge conducted 50 days after the initiation of specific diets with different concentrations of testers). Hemagglutinin titers were enhanced in mice when supplemented with either ester and the effects were dose-dependent. Vitamin E facilitated a shift from IgM to IgG antibody production, causing the authors to hypothesize that the supplementation enhanced the helper T-cell activity.

## 9. Cancer

Prasad et al. [50] provided a review of α-tocopheryl succinate as a potential adjuvant cancer treatment. This review provided a history of the discovery of the anticancer activity of the succinate ester, a discussion of the possible mechanisms of this activity, and a discussion of its activity in combination with radiation or chemotherapeutics. In this review, authors referenced a previous study that demonstrated that α-tocopheryl succinate, but not α-tocopheryl acetate or α-tocopheryl nicotinate, induces differentiation, inhibits proliferation, and promotes cell death in murine melanoma cells. In an earlier study, Sahu et al. [51] demonstrated that the mechanism by which α-tocopheryl succinate inhibits tumor growth is by decreasing melanocyte-stimulating hormone adenylate cyclase activity. None of the other esters of tocopherol had such an effect on melanocyte stimulating hormone-stimulated adenylate cyclase activity. In short, α-tocopheryl nicotinate is not implicated in vitamin E adjuvant cancer treatment; solely the succinate ester appears to have anticancer activity.

## 10. Conclusions

The characteristics and medical uses of vitamin E are well characterized in the scientific literature. Most studies, in vitro and in vivo, of the effects of vitamin E utilize the α-tocopheryl acetate ester as the form of vitamin E administered. By comparison, the literature referencing α-tocopheryl nicotinate specifically is sparse and outdated. However, some differential characteristics and effects have been identified (Table 2) and α-tocopheryl nicotinate has been patented directly for use in diabetes, hypertension, the inhibition of viral replication, and reducing body fat. Most of the differential effects of α-tocopheryl nicotinate in vivo appear to be the result of slower hydrolysis and greater absorption in the esterified form. More in vivo comparison studies against other esters of tocopherol and multigroup clinical trials would be required to establish if the differential effects of α-tocopheryl nicotinate versus other tocopherol esters are clinically relevant and to elucidate further the mechanisms of α-tocopheryl nicotinate exceptionalism. Further, endogenous α-tocopheryl nicotinate may have pathophysiological importance. We hypothesize that this endogenous α-tocopheryl nicotinate is formed by the re-esterification of α-tocopherol derived from the diet with free niacin. Further studies are required to identify the mechanisms of this re-esterification.

## Figures and Tables

**Table 1:** Reported Frequencies of Uses of Vitamin E Derivatives in Cosmetic Formulations [15].

Ranking	Vitamin E Derivative	Frequency of Use
1	Tocopheryl acetate	1322
2	Tocopherol	1072
3	Tocopheryl linoleate	279
4	Potassium ascorbyl tocopheryl phosphate	15
5	Dioleyl tocopheryl methylsilanol	12
6	Tocopheryl succinate	4
7	Tocopheryl nicotinate	3
8	Tocophersolan	2

**Table 2:** Summary of the studies on α-tocopheryl nicotinate.

Reference	Test	Object	Subjects	αTN Dose	Duration	Design	Control	Method	Conclusion
Koyama & Araiso [28]	Rheological properties	Erythrocytes	7 healthy human patients	400 mg/day	1 month	Paired	Untreated baseline	Oral at mealtime	Decrease in membrane viscosity
Chung et al. [29]	Retinal blood flow	Blood viscosity, composition	7 female diabetes patients	900 mg/day	3 months	Paired	Untreated baseline	Oral at mealtime	Improved red blood cell deformity
Chung et al. [30]	Rheological properties	Erythrocytes	13 type II diabetic patients w/ retinopathy	900 mg/day	3 months	Paired	Untreated baseline	Oral at mealtime	Reduction in blood viscosity & red blood cell oxidation
Kamimura [32]	Microcirculation	Mean rewarming time (MRT)	36 microcirculatory deficiency patients	400 mg/day	2 weeks	Paired, cross administration	αTA & nicotinic acid	Oral at mealtime	αTN superior to αTA in reducing MRT
Kamimura [31]	Microcirculation	Mean rewarming time (MRT)	10 microcirculatory deficiency patients	400 mg/day	2 weeks	Paired, cross administration	αTA & nicotinic acid	Oral at mealtime	αTN superior to αTA in reducing MRT
Igarishi et al. [38]	Hypertension	Blood pressure, animal weight	SHR and DOCA-salt hypertensive rats	100 mg/kg/day	4 weeks	Treated vs. controls	Gum arabic solution	Oral gavage once daily	Systolic blood pressure reduced by 15% compared to controls
Iino et al. [39]	Hypertension	Subjective symptoms	89 patients with hypertension or arteriosclerosis	600 mg/day	4–6 weeks	Treated vs. controls	Placebo	Oral at mealtime	Symptoms improved with αTN
Hidiroglou et al. [40]	Cholesterol, HDL	Blood concentrations	40 wether lambs	300 mg/day	8 weeks	Treated vs. controls	Placebo	Mixed with commercial diet	No significant effects on cholesterol or HDL levels
Higashi & Kikuchi [41]	Platelet aggregation	Platelet-rich plasma	in vitro	0.1–0.25 mM	1 h	Treated vs. controls	αTA	3uL in vitro	αTN superior to αTA in reducing hydrogen peroxide-induced platelet aggregation
Svensson & Oki [42]	Platelet aggregation	Platelet-rich plasma	in vitro	200 μg/mL	2–3 h	treated vs. controls	α-Tocopherol and αTA	in Vitro bath	αTN 18x more potent than αT and 5x more potent than αTA at inhibiting platelet aggregation due to arachidonic acid
Noma et al. [43]	Atherogenesis	Serum lipoprotein(a)	28 Hyperlipidemic patients	600 mg/day	2 months	Paired	Untreated baseline	Oral at mealtime	Lipoprotein(a) concentrations declined significantly in patients with initial lipoprotein(a) concentrations >18 mg/dL
Schlieper & Tawfil [18]	Arrhythmias	Inotropic action of glycosides	Guinea pigs atria	100 μM	1 h	Treated vs. controls	Ethanol, dodecanoic acid, α-tocopherol	in vitro bath	αTN more potent than α-tocopherol and dodecanoic acid in supressing inotropic effect of digoxin but not ouabain and also results in >90% decrease in arrhythmic activity of glycosides
Moriguchi & Itoh [47]	Immune system	T-cell differentiation	Male Fischer rats	585 mg/kg/day	7 weeks	High vs. low αTN	Low-αTN diet rats	Mixed with commercial diet	Interleukin 2 production increased and PGE2 production decreased in thymocytes and CD4^+^ cells increased in rats fed high αTN diet
Inagaki et al. [48]	Immune system	IgE antibody generation	Female BALB/c mice, male Wistar rats	226 mg/kg food	4 weeks	Treated vs. controls	Low vitamin E diets, αTA	Mixed with commercial diet	αTN more potent than αTA in suppressing IgE production and stimulating non-IgE antibody in antigen challenge studies
Tanaka et al. [49]	Immune system	Humoral immune response	Female SL and DDD mice	226 mg/kg food	50 days	Treated vs. controls	Low vitamin E diets, αTA	Mixed with commercial diet	αTA diet more potent than αTN diet in enhancing humoral immune response to antigen challenge; neither αTA nor αTN produced a significant effect
Prasad et al. [50]	Cancer	Melanoma cell	murine melanoma (B-16) and fibroblast (L-cells) cells	1–100 μg/mL	2 days	Treated vs. controls	Free alcohol, αTA, αTS	in vitro bath	αTS inhibiting melanoma cell proliferation; αTN and αTA not suppressing melanoma proliferation

Abbreviations: αTN, α-tocopheryl nicotinate; αTA, α-tocopheryl acetate; αTS, α-tocopheryl succinate.
