# Correction: Duncan, K.R., Suzuki, Y.J. Vitamin E Nicotinate. *Antioxidants* 2017, *6*, 20

**DOI:** 10.3390/antiox7050064

**Published:** 2018-05-03

**Authors:** Kimbell R. Duncan, Yuichiro J. Suzuki

**Affiliations:** Department of Pharmacology and Physiology, Georgetown University Medical Center, 3900 Reservoir Road NW, Washington, DC 20057, USA; krd49@georgetown.edu

In the original version of our article [1], three lines were omitted from the α-tocopherol and α-tocopheryl nicotinate structures in Figure 1 during the manuscript processing. The authors wish to make the following correction and replace Figure 1. 
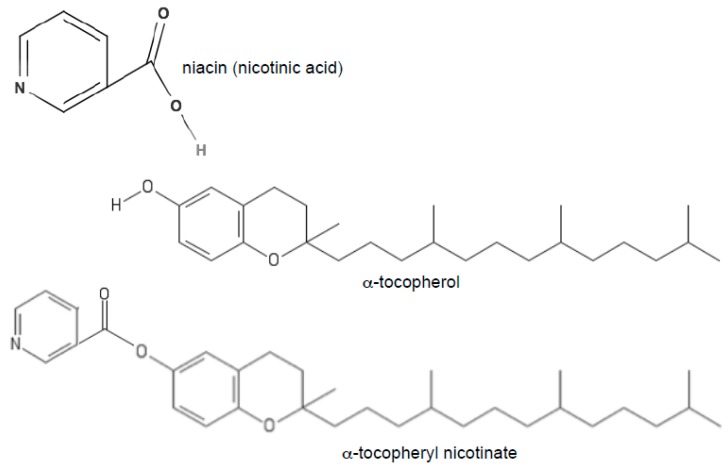
 with: 
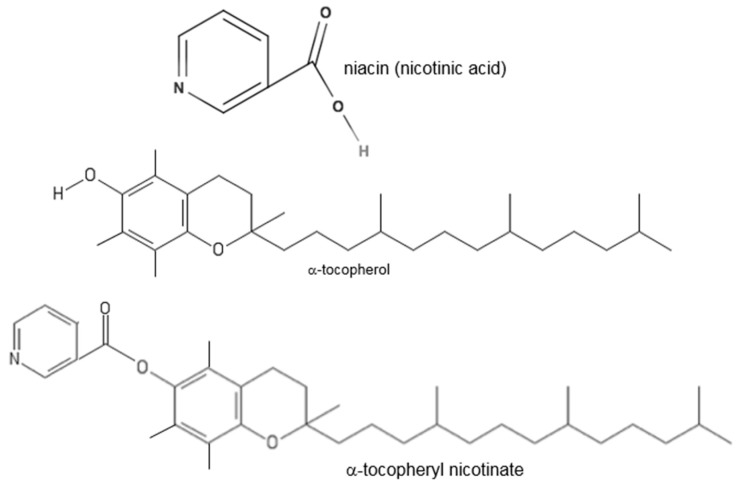


The authors would like to apologize for any inconvenience caused. The change does not affect the scientific results. The manuscript will be updated and the original will remain online on the article webpage.
